# Use of folic acid and vitamin supplementation among adults with depression and anxiety: a cross-sectional, population-based survey

**DOI:** 10.1186/1475-2891-10-102

**Published:** 2011-09-30

**Authors:** Guixiang Zhao, Earl S Ford, Chaoyang Li, Kurt J Greenlund, Janet B Croft, Lina S Balluz

**Affiliations:** 1 Division of Adult and Community Health, National Center for Chronic Disease Prevention and Health Promotion, Centers for Disease Control and Prevention, Atlanta, Georgia, USA

**Keywords:** folic acid, vitamins, depression, anxiety, elevated depressive symptoms, BRFSS

## Abstract

**Background:**

Evidence suggests that folate deficiency may be causatively linked to depressive symptoms. However, little is known on the status of use of folic acid and vitamin supplements among people with mental disorders. This study examined the prevalence and the likelihood of use of folic acid or vitamin supplements among adults with depression and anxiety in comparison to those without these conditions.

**Methods:**

Using data from 46, 119 participants (aged ≥ 18 years) in the 2006 Behavioral Risk Factor Surveillance System survey, we estimated the adjusted prevalence and odds ratios with 95% confidence intervals for taking folic acid and vitamin supplements among those with ever diagnosed depression (n = 8, 019), ever diagnosed anxiety (n = 5, 546) or elevated depressive symptoms (n = 3, 978, defined as having a depression severity score of ≥ 10 on the Patient Health Questionnaire-8 diagnostic algorithm).

**Results:**

Overall, women were more likely than men to take folic acid supplements 1-4 times/day (50.2% versus 38.7%, P < 0.001) and vitamin supplements (62.5% versus 49.8%, P < 0.001). After multivariate adjustment, men with ever diagnosed depression or anxiety were 42% and 83%, respectively, more likely to take folic acid supplements < 1 time/day; 44% and 39%, respectively, more likely to take folic acid supplements 1-4 times/day; and 40% and 46%, respectively, more likely to take vitamin supplements compared to men without these conditions (P < 0.05 for all comparisons). Women with ever diagnosed depression were 13% more likely to take folic acid supplements 1-4 times/day and 15% more likely to take vitamin supplements than women without this condition (P < 0.05 for both comparisons). Use of folic acid and vitamin supplements did not differ significantly by elevated depressive symptoms in either sex.

**Conclusion:**

The prevalence and the likelihood of taking folic acid and vitamin supplements varied substantially by a history of diagnosed depression among both men and women and by a history of diagnosed anxiety among men, but not by presence of elevated depressive symptoms in either sex.

## Background

Depression is recognized as an important cause of disability and mortality worldwide [1,2]. Data from a population-based survey in the United States showed that, in 2006, approximately 15.4% of adults aged ≥ 18 years reported a history of diagnosed depression, 8.4% reported having moderate-to-severe depressive symptoms in the previous 2 weeks, and 11.0% reported a history of diagnosed anxiety [3]. The burden of depression and other mental disorders in the United States is substantial and associated with a cost of $150 billion each year [4].

Evidence suggests that folate deficiency is causatively linked to depressive symptoms because folate plays an important role in the one-carbon metabolic pathway involved in methylation processes and the synthesis of neurotransmitters in the central nervous system [5,6]. Elevated homocysteine levels, a marker of folate deficiency as well as vitamin B12 deficiency, can also cause oxidative stress, resulting in cerebral vascular and neurological damage and neurotransmitter deficiency [7,8]. To date, majority of cross-sectional studies, several prospective studies, and meta-analyses have shown that low levels of serum or red blood cell folate, low levels of serum vitamin B12, low dietary intake of folate and vitamin B12, and high levels of serum homocysteine are associated with an increased risk for depression [9-28]. Low folate levels have also been associated with severe depressive disorders and longer duration of depressive episodes [29]. In addition, depressed people with low serum folate levels are significantly less likely to respond to some antidepressant medications (such as fluoxetine) [30-32] and more likely to relapse during treatment [33]. In contrast, higher intakes of folate, vitamins B6 and B12 have been shown to be protective of depressive symptoms in some studies [34-36], but not in others [37].

The National Health and Nutrition Examination Survey (NHANES) data from 1999-2000 showed that 52% of U.S. adults reported taking dietary supplements in the previous month and that 35% reported use of multivitamin-multimineral supplements [38]. However, the status of use of folic acid and vitamin supplements among people with depression is unknown. We hypothesized that use of these supplements may be positively associated with previously diagnosed depression; the currently elevated depressive symptoms may be inversely related to use of these supplements. To shed light on these questions, we examined the prevalence and the likelihood of taking folic acid and vitamin supplements among adults with depressive disorders in comparison with those without these conditions by using data from a population-based survey sample. In addition, because depression and anxiety often coexist in people with mental disorders [39], we also examined the prevalence and the likelihood of taking folic acid and vitamin supplements among adults with or without ever diagnosed anxiety, although associations of folate and B-vitamins with anxiety remain to be elucidated.

## Methods

### Survey design, participants, and data

Data for our analyses came from the Behavioral Risk Factor Surveillance System (BRFSS), a population-based telephone survey of health-related behaviors regarding the leading causes of death among noninstitutionalized U.S. adults aged ≥ 18 years. The BRFSS is considered to be exempt from review by the Institutional Review Board of the Centers for Disease Control and Prevention. The BRFSS survey design, sampling methods and weights have been described elsewhere [40], and BRFSS data have consistently been found to provide valid and reliable estimates when compared with data from other national household surveys in the United States [40-42]. Further information on the BRFSS is available at http://www.cdc.gov/brfss/.

We analyzed the data collected from survey participants in 9 states (Delaware, Florida, Georgia, Minnesota, Missouri, Montana, Virginia, Wisconsin, and Wyoming) and Puerto Rico that had optional modules on folic acid and vitamin supplementation and on anxiety and depression in the 2006 BRFSS. The median cooperation rate (the percentage of eligible persons contacted who completed the interview) for the 2006 BRFSS was 74.5%.

Use of vitamin supplements was assessed by asking participants whether they were currently taking any vitamin pills or supplements and their response to the question was dichotomized as yes/no. The use of folic acid supplements was assessed by asking participants whether they were taking folic acid supplements or taking multivitamins or any vitamin pills/supplements that contained folic acid and, if so, how many times a day they were taking such supplements. Their responses to the question were categorized as 1) not taking folic acid supplements, 2) taking folic acid supplements < 1 time/day, and 3) taking folic acid supplements 1-4 times/day. Those who answered that they were taking folic acid supplements > 4 times/day were excluded from the analyses because fewer than 0.5% of participants were in this category. Self-reports of vitamin and mineral supplement use have been reported to have high reliability and validity [43].

Participants' mental health status was assessed by asking them whether they had ever been told by a doctor or other healthcare provider that they had a depressive disorder (including depression, major or minor depression, or dysthymia) or an anxiety disorder (including acute or posttraumatic stress disorder, anxiety, generalized anxiety disorder, obsessive-compulsive disorder, panic disorder, phobia, or social anxiety disorder). Those who answered "yes" to either question were defined as having ever diagnosed depression or anxiety. Participants' current depressive symptoms were assessed using the Patient Health Questionnaire-8 (PHQ-8) diagnostic algorithm, which has been described in detail elsewhere [3,44,45]. Specifically, participants were asked about how many days in the previous 2 weeks they experienced each of the following symptoms of depression: 1) little interest or pleasure in doing things; 2) feeling down, depressed, or hopeless; 3) having trouble falling asleep or staying asleep or sleeping too much; 4) feeling tired or little energy; 5) having a poor appetite or eating too much; 6) feeling bad as a failure or letting themselves or their family down; 7) having trouble concentrating on things such as reading the newspaper or watching the TV; and 8) moving or speaking too slowly or being so fidgety or restless that they were moving around a lot more than usual. For each item, respondents scored as 0 point for 0-1 day with reported symptoms, 1 point for 2-6 days, 2 points for 7-11 days, and 3 points for 12-14 days. Their scores for each item were then added to produce a total depression severity score, and those with a total depression severity score of ≥ 10 were defined as having elevated depressive symptoms (EDS). The PHQ-8 has been shown to provide valid measurements on major depression in the general population similar to that of the PHQ-9 (which includes the ninth question about thoughts of suicidality or self-injury) [46-49], and the scoring of ≥ 10 on the PHQ-8 has been shown to have a sensitivity and specificity of 88% for major depression [47]. Currently, the reliability or validity of the measures for physician diagnosis of depression and anxiety in the BRFSS has not been determined due to lack of research. However, in psychiatric epidemiology, lifetime prevalence, estimated as the proportion of a sample having had at least one episode of mental disorders in their life prior to the survey, is one of the most commonly used parameters to assess history of mental disorders [3,39,44,45]. This measure reflects a view that most mental disorders are chronic conditions and symptomatic episodes of mental disorders are often interspersed with periods of remission.

The demographic variables in our analyses included respondents' age, sex, race/ethnicity (non-Hispanic white, non-Hispanic black, Hispanic, or other), education (< high school diploma, high school graduate, some college or technical school, or ≥ college graduate), marital status (married, divorced, never married, or other), and body mass index (BMI, self-reported weight divided by height in square). Respondents' smoking status was categorized as current smokers (participants who had smoked ≥ 100 cigarettes during their lifetime and were still smoking), former smokers (those who had smoked ≥ 100 cigarettes in their entire life but had stopped smoking), and never smokers (those who had never smoked or smoked < 100 cigarettes during their lifetime). Leisure-time physical activity was assessed by asking respondents whether, during the previous month, they had participated in any physical activities or exercise other than their regular job. Heavy alcohol drinking was defined as women who had > 1 drink/day or men who had > 2 drinks/day during the previous 12 months.

### Statistical analyses

Of a total of 55, 867 survey participants, after excluding from the analyses participants who responded "don't know/not sure, " refused to answer, or had missing responses for any of the questions described above, 46, 119 participants remained in our analyses. We estimated the adjusted prevalence of taking folic acid and vitamin supplements in adult men and women. We tested the interactive effects of sex with all three mental disorders on the outcome measurements (i.e., use of folic acid and vitamin supplements) in the fully adjusted models which included the main effects of sex and mental disorders. The interaction terms were significant so we conducted sex-stratified analysis. Adjusted prevalence (predicted marginal probability) of taking folic acid and vitamin supplements was estimated by conducting logistic regression analyses after adjustment for age, race/ethnicity, education, body mass index, marital status, smoking status, leisure-time physical activity, and heavy alcohol drinking. We also estimated the adjusted odds ratios (AORs) with 95% confidence intervals (CIs) for taking folic acid and vitamin supplements using mental disorders as exposure variables while controlling for demographic variables and lifestyle factors as listed above. We used SUDAAN, 9.0 (Research Triangle Institute, Research Triangle Park, NC) to account for the multistage, disproportionate stratified sampling design.

## Results

Of the 46, 119 participants, the age-adjusted prevalence of ever diagnosed depression was 15.0% [men-10.4% versus women-19.4% (P < 0.001)], the age-adjusted prevalence of ever diagnosed anxiety was 11.2% [men-8.2% versus women-14.1% (P < 0.001)], and the age-adjusted prevalence of EDS was 8.3% [men-6.5% versus women-10.0% (P < 0.001)]. The mean age of participants was 45.8 years, and the mean BMI was 27.1 kg/m^2^. Approximately 49.2% of participants were men, 61.6% were married, and 34.6% had an education of ≥ college graduate. By race/ethnicity, 70.9% were non-Hispanic white, 9.5% were non-Hispanic black, 14.7% were Hispanic, and 4.9% were of "other" race/ethnicity.

Participants with any of the three categories of mental disorder were significantly younger than those without (P < 0.001 for all) (Table 1). The weighted percentages of participants who were male, married, or ≥ college graduates were significantly lower (P < 0.01 for all), and the weighted percentages of adults who were obese were significantly higher (P < 0.001), among those with mental disorders than among those without these conditions (Table 1).

**Table 1:** Demographic characteristics of study population (N = 46, 119) by depression and anxiety status, BRFSS, 2006*

	Ever diagnosed depression		Ever diagnosed anxiety		EDS	
			
	yes	no	yes	no	yes	no
	n = 8, 019	n = 38, 100	n = 5, 546	n = 40, 573	n = 3, 978	n = 42, 141
Age (years)	44.4 (0.3)	46.0 (0.2)	43.7 (0.4)	46.0 (0.2)	42.0 (0.5)	46.1 (0.2)
Sex						
Men	34.4 (1.0)	51.8 (0.4)	36.1 (1.2)	50.9 (0.4)	38.8 (1.6)	50.2 (0.4)
Women	65.6 (1.0)	48.2 (0.4)	63.9 (1.2)	49.1 (0.4)	61.2 (1.6)	49.8 (0.4)
Race/ethnicity						
Non-Hispanic white	75.1 (0.8)	70.2 (0.4)	72.0 (1.0)	70.8 (0.4)	64.4 (1.5)	71.5 (0.4)
Non-Hispanic black	6.8 (0.6)	10.0 (0.3)	7.2 (0.7)	9.8 (0.3)	11.5 (1.0)	9.3 (0.2)
Hispanic	13.4 (0.6)	14.9 (0.3)	15.5 (0.8)	14.6 (0.3)	18.0 (1.2)	14.4 (0.3)
Others	4.8 (0.4)	4.9 (0.2)	5.2 (0.5)	4.8 (0.2)	6.1 (0.7)	4.8 (0.2)
Education						
< high school diploma	11.4 (0.7)	9.2 (0.2)	13.0 (0.8)	9.1 (0.2)	21.1 (1.3)	8.5 (0.2)
High school graduate	29.1 (0.9)	28.1 (0.4)	28.8 (1.1)	28.2 (0.4)	33.5 (1.4)	27.8 (0.4)
Some college/technical	29.4 (0.9)	27.3 (0.4)	29.2 (1.1)	27.4 (0.4)	27.9 (1.4)	27.6 (0.4)
≥ College graduate	30.1 (0.9)	35.4 (0.4)	29.0 (1.0)	35.3 (0.4)	17.5 (1.1)	36.1 (0.4)
Body mass index (kg/m^2^)						
< 25.0	36.1 (1.0)	38.7 (0.4)	37.7 (1.2)	38.4 (0.4)	34.7 (1.5)	38.6 (0.4)
25.0- < 30.0	31.7 (0.9)	37.7 (0.4)	33.1 (1.1)	37.2 (0.4)	29.5 (1.4)	37.4 (0.4)
≥ 30.0	32.2 (0.9)	23.7 (0.4)	29.2 (1.0)	24.4 (0.4)	35.8 (1.3)	24.0 (0.3)
Marital status						
Married	50.7 (1.0)	63.5 (0.4)	51.0 (1.2)	62.9 (0.4)	42.4 (1.4)	63.3 (0.4)
Divorced	15.0 (0.6)	8.0 (0.2)	14.1 (0.6)	8.4 (0.2)	14.8 (0.8)	8.5 (0.2)
Never married	18.4 (1.0)	17.4 (0.4)	20.2 (1.2)	17.2 (0.4)	23.0 (1.6)	17.1 (0.4)
Others	15.8 (0.7)	11.1 (0.2)	14.7 (0.8)	11.5 (0.2)	19.8 (1.3)	11.1 (0.2)

* Data presented as weighted percentages with standard errors in parentheses except for mean age. BRFSS = Behavioral Risk Factor Surveillance System, EDS = Elevated depressive symptoms

Overall, after multivariate adjustment for age, race/ethnicity, education, marital status, BMI, smoking status, leisure-time physical activity, and heavy alcohol drinking, women had a significantly higher prevalence of taking folic acid supplements 1-4 times/day (50.2% versus 38.7%, P < 0.001; AOR:1.74, 95% CI:1.63-1.86) or taking vitamin supplements (62.5% versus 49.8%, P < 0.001; AOR:1.76, 96% CI:1.64-1.89) than men. In our logistic regression models, we stratified data analyses by sex because of the significant interactive effects of sex and mental disorders on the outcome measurements. Compared with men without diagnosed depression and anxiety, those with a history of these diagnoses had a significantly higher prevalence of taking folic acid supplements 1-4 times/day, and significantly higher AORs for taking folic acid supplements at both levels (< 1 time/day or 1-4 times/day) (P < 0.05 or P < 0.001, Table 2). Women with ever diagnosed depression were also significantly more likely than women without this condition to take folic acid supplements 1-4 times/day (52.7% versus 49.8%, P < 0.05; AOR: 1.13, 95% CI: 1.02-1.26). Similarly, men with ever diagnosed depression (56.5% versus 48.8%, P < 0.001; AOR: 1.40, 95% CI: 1.18-1.66) or anxiety (57.5% versus 49.0%, P < 0.001; AOR: 1.46, 95% CI: 1.18-1.80) and women with ever diagnosed depression (65.0% versus 62.0%, P < 0.01; AOR: 1.15, 95% CI: 1.04-1.28) were significantly more likely to report taking vitamin supplements than those who had never been diagnosed with these conditions after adjustment for multiple demographic variables and lifestyle factors (Table 3). No significant associations of EDS with taking folic acid and vitamin supplements were observed in either sex (Table 2 and Table 3).

**Table 2:** Adjusted prevalence (with standard error) of and adjusted odds ratios (with 95% confidence intervals) for taking folic acid supplements among adults aged ≥ 18 years, by sex and by depression and anxiety status, BRFSS, 2006

	Adjusted prevalence*			Adjusted odds ratio*			
		
	Taking supplements		No supplements	Taking supplements			
		
(times/day)	< 1	1 to 4		< 1		1 to 4	
	**% (SE)**	**% (SE)**	**% (SE)**	**AOR**	**95% CI**	**AOR**	**95% CI**
**Men (n = 18, 092)**							
Ever diagnosed depression							
Yes (n = 2, 140)	7.6 (1.1)	45.1 (1.8)¶	47.3 (1.9)¶	1.42	1.00-2.00	1.44	1.21-1.71
No (n = 15, 952)	6.4 (0.4)	38.0 (0.6)	55.6 (0.6)	1.00		1.00	
Ever diagnosed anxiety							
Yes (n = 1, 508)	9.6 (1.8)†	43.6 (2.2)‡	46.8 (2.3)¶	1.83	1.18-2.84	1.39	1.13-1.70
No (n = 16, 584)	6.3 (0.4)	38.3 (0.6)	55.4 (0.6)	1.00		1.00	
EDS							
Yes (n = 1, 162)	10.6 (2.7)	37.5 (2.4)	52.0 (3.1)	1.79	0.96-3.34	1.02	0.80-1.30
No (n = 16, 930)	6.3 (0.4)	38.8 (0.6)	54.9 (0.6)	1.00		1.00	
**Women (n = 28, 027)**							
Ever diagnosed depression							
Yes (n = 5, 879)	6.6 (0.6)	52.7 (1.1)‡	40.7 (1.0)‡	0.97	0.76-1.24	1.13	1.02-1.26
No (n = 22, 148)	7.2 (0.3)	49.8 (0.5)	43.0 (0.5)	1.00		1.00	
Ever diagnosed anxiety							
Yes (n = 4, 038)	7.0 (0.8)	51.8 (1.3)	41.3 (1.2)	1.02	0.78-1.33	1.08	0.96-1.22
No (n = 23, 989)	7.1 (0.3)	50.1 (0.5)	42.8 (0.5)	1.00		1.00	
EDS							
Yes (n = 2, 816)	7.5 (1.2)	49.2 (1.6)	43.2 (1.5)	1.05	0.73-1.51	0.96	0.83-1.10
No (n = 25, 211)	7.0 (0.3)	50.5 (0.5)	42.5 (0.5)	1.00		1.00	

*Adjusted for age, race/ethnicity, education, body mass index, marital status, smoking, leisure-time exercise, and alcohol consumption. †P = 0.065, ‡P < 0.05, and ¶P < 0.001 for comparisons between adults with ever diagnosed depression or anxiety and those without. AOR = Adjusted odds ratio, BRFSS = Behavioral Risk Factor Surveillance System, CI = Confidence interval, EDS = Elevated depressive symptoms, SE = Standard error

**Table 3:** Adjusted prevalence (with standard error) of and adjusted odds ratios (with 95% confidence intervals) for taking vitamin supplements among adults aged ≥ 18 years, by sex and by depression and anxiety status, BRFSS, 2006

	Adjusted prevalence*	Adjusted odds ratio*	
		
	% (SE)	AOR	95% CI
**Men (n = 18, 497)**			
Ever diagnosed depression			
Yes (n = 2, 140)	56.5 (1.8)¶	1.40	1.18-1.66
No (n = 15, 952)	48.8 (0.6)	1.00	
Ever diagnosed anxiety			
Yes (n = 1, 508)	57.5 (2.3)¶	1.46	1.18-1.80
No (n = 16, 584)	49.0 (0.6)	1.00	
EDS			
Yes (n = 1, 162)	52.1 (3.1)	1.12	0.86-1.47
No (n = 16, 930)	49.5 (0.6)	1.00	
**Women (n = 28, 332)**			
Ever diagnosed depression			
Yes (n = 5, 879)	65.0 (1.0)§	1.15	1.04-1.28
No (n = 22, 148)	62.0 (0.5)	1.00	
Ever diagnosed anxiety			
Yes (n = 4, 038)	64.0 (1.2)	1.08	0.95-1.22
No (n = 23, 989)	62.4 (0.5)	1.00	
EDS			
Yes (n = 2, 816)	61.2 (1.5)	0.92	0.80-1.07
No (n = 25, 211)	62.8 (0.5)	1.00	

*Adjusted for age, race/ethnicity, education, body mass index, marital status, smoking, leisure-time physical activity, and alcohol consumption. § P < 0.01, ¶ P < 0.001 for comparisons between adults with ever diagnosed depression or anxiety and those without. AOR = Adjusted odds ratio, BRFSS = Behavioral Risk Factor Surveillance System, CI = Confidence interval, EDS = Elevated depressive symptoms, SE = Standard Error

**Figure 1:**
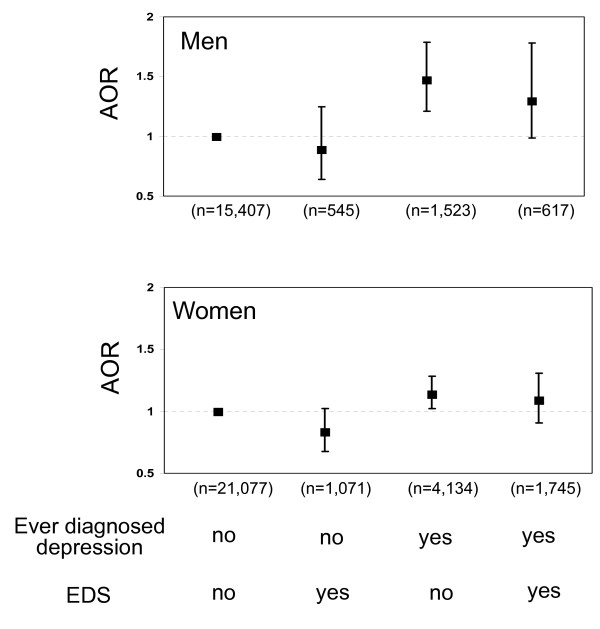
**Adjusted odds ratios (with 95% confidence intervals) for taking folic acid supplements 1-4 times/day among men (top) and women (bottom) aged ≥ 18 years with either ever diagnosed depression or elevated depressive symptoms (EDS) or both**.

**Figure 2:**
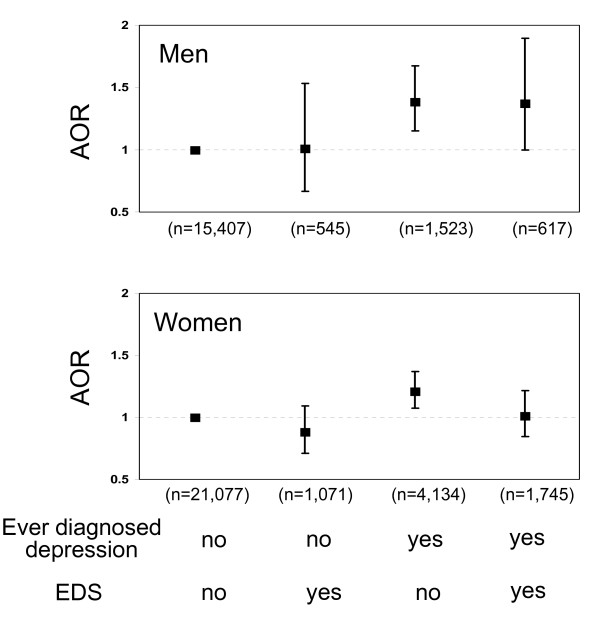
**Adjusted odds ratios (with 95% confidence intervals) for taking vitamin supplements among men (top) and women (bottom) aged ≥ 18 years with either ever diagnosed depression or elevated depressive symptoms (EDS) or both**.

For depression status, people who were ever diagnosed with depression may or may not have EDS currently --this may have affected their behaviors of taking dietary supplements. Thus, we conducted further stratified analysis by ever diagnosed depression and/or EDS. A total of 5, 657 participants [the age-adjusted prevalence: total-10.6%; men-7.4% versus women-13.6% (P < 0.001)] reported having ever diagnosed depression only, 1, 616 [the age-adjusted prevalence: total-3.9%; men-3.4% versus women-4.3% (P < 0.05)] reported having EDS only, and 2, 362 [the age-adjusted prevalence: total-4.4%; men-3.1% versus women-5.6% (P < 0.001)] reported having both. Compared with men having neither ever diagnosed nor EDS, those with ever diagnosed depression, regardless whether they had EDS currently, were significantly more likely to take folic acid supplements 1-4 times/day (AOR: 1.48, 95% CI: 1.22-1.81 for those with ever diagnosed depression only and AOR: 1.31, 95% CI: 0.99-1.80 for those with both ever diagnosed depression and EDS) and to take vitamin supplements (AOR: 1.40, 95% CI: 1.16-1.69 for those with ever diagnosed depression only and AOR: 1.39, 95% CI: 1.00-1.92 for those with both ever diagnosed depression and EDS) (Figure 1 and Figure 2). Women with ever diagnosed depression but without EDS were also significantly more likely to take folic acid supplements 1-4 times/day (AOR: 1.13, 95% CI: 1.01-1.28) and to take vitamin supplements (AOR: 1.20, 95% CI: 1.06-1.36) than were women with neither conditions (Figure 1 and Figure 2); however, these associations were not significant in women with both ever diagnosed depression and EDS.

## Discussion

Our results showed that, among participants from 9 states and 1 territory of the United States, use of folic acid supplements 1-4 times/day (50.2% versus 38.7%) and use of vitamin supplements (62.5% versus 49.8%) were significantly higher among women than among men. Men with ever diagnosed depression were more likely to report taking folic acid and vitamin supplements than men with no history of diagnosed depression regardless whether they had elevated depressive symptoms. In contrast, although women with ever diagnosed depression were significantly more likely to report taking folic acid 1-4 times/day and vitamin supplements than were women with no history of diagnosed depression, these differences were no longer statistically significant when the status on elevated depressive symptoms was taken into consideration.

To date, most previous studies have mainly focused on the causal relationship between folate and mental disorders. The results of these studies suggest that folate or folinic acid (5-methyltetrahydrofolate) may serve as a stand-alone treatment of depression [50,51] or an augmentation of antidepressant treatment showing a significant beneficial effect in reducing Hamilton Depression Rating Scale Score when used with other psychotropic medication [52-57]. Limited evidence from the Vitamins and Lifestyle (VITAL) study showed that 10.4% of participants with depression who were taking medications for depression reported taking dietary supplements and that 6.7% of those who felt depressed or anxious but did not take medication for depression reported doing so [58]. In that study, participants were limited to the 50 to 75 years of age, and dietary supplements were a combination of 17 supplements including folic acid and various other vitamins. To our knowledge, our study is the first population-based study to focus on the behaviors of use of folic acid and vitamin supplements among adults with depression or anxiety. Although our results showed that men with ever diagnosed depression or anxiety and women with ever diagnosed depression were more likely than those without a history of these conditions to report taking folic acid supplements (1-4 times/day), only about 50% of them were doing so. Most importantly, we found that adults with elevated depressive symptoms were only as likely as those without these symptoms to report taking folic acid supplements. At present, studies have consistently reported a high prevalence of depressive symptoms in populations [3,45,59], and these symptoms are strongly associated with first-onset major depression [59]. In addition, most depressive symptoms are often unrecognized and untreated in clinical practice. Given the links between folate deficiency and depressive symptoms [9-28], the use of folic acid supplements may possibly prevent or delay the onset of major depression among people with elevated depressive symptoms, although randomized trials are needed to confirm it.

Other than folate, studies have shown that low levels of vitamin B6 and B12 were also related to depressive disorders [16,19,23,26,27,60-62], however, other studies failed to show any relationship between vitamin B12 and depressive symptoms [12,20,25,30]. Similarly, high intakes of vitamins B6 and B12 have shown to be protective of depressive symptoms in a prospective study [36], but not in others [34,37]. Thus, the controversial results remained to be elucidated in the future. Nonetheless, B-vitamins including B6 and B12 have known to be important for essential functioning of the one-carbon metabolism in the biosynthesis of monoamine neurotransmitters including serotonin, dopamine, norepinephrine, and epinephrine [60-62], all of which are known to affect mood and cognition. Our results showed that, although adults with ever diagnosed depression were significantly more likely to report taking vitamin supplements than those with no history of diagnosed depression, only less than two-thirds of them reported taking vitamin supplements. Taken together, our results call for further research or clinical trials that may help to establish whether the use of folic acid and vitamin supplements should be improved among people with mental disorders.

Our results further demonstrated that men with ever diagnosed anxiety were significantly more likely to report taking folic acid and vitamin supplements, although these relationships were not observed among women with anxiety. At present, the relationship between anxiety and use of folic acid and vitamin supplementats is unknown. However, given the high prevalence of anxiety in the U.S. population [3,45] and its coexistence with depression [39], further investigation of the role of folic acid and vitamin supplementation in the prevention and treatment of anxiety is needed.

There are several limitations in the present study. First, all data were based on the self-reports by survey participants and thus subject to recall bias. Second, the information on the severity and diagnosis date of mental disorders was not available in the BRFSS. Third, given the cross-sectional nature of the survey, the temporal relationship between the use of folic acid and vitamin supplements and the presence of depression, anxiety, or elevated depressive symptoms cannot be established in the present study. Evidence suggests that folate/vitamin deficiency may affect one-carbon metabolic pathway and neurotransmitter synthesis in the central nervous system, thereby linking to depressive disorders. On the other hand, people who are diagnosed with mental disorders may be more likely to take dietary supplements. Thus, bidirectional associations may exist. Fourth, although we have assessed the frequency of folic acid supplementation, the amount of daily folic acid and vitamin intake as well as the biomarkers of folic acid and vitamin supplementation (i.e., serum or red blood cell folate levels, serum homocysteine levels, or serum vitamin levels) was unknown in the present study. Also, data on dietary intake of folate and vitamins as well as dietary energy intake were not available in the BRFSS so we were unable to assess whether these variables may have confounding effects. Fifth, women of reproductive age may be more likely to take folic acid and vitamin supplements. In addition, people with chronic conditions such as cardiovascular disease and diabetes may confer higher risks for depression. Thus, further stratified analysis in women of childbearing age or in people with chronic conditions are warranted in future studies. Finally, use of prescribed medications (including prescribed folate or other supplements that contain folate) for depression, anxiety, or other mental disorders was not ascertained, thus, we were unable to conduct stratified analyses by medication use.

## Conclusions

Our results from a large, nationally representative sample demonstrated that substantial variations exist in the prevalence and the likelihood of taking folic acid and vitamin supplements by sex and by histories of previously diagnosed depression and anxiety, but not by presence of elevated depressive symptoms. Our results provide fundamental information on the status of dietary supplement use among U.S. adults with mental disorders, and have important implications in public health nutrition given that high intakes of folic acid and vitamins may reduce the risk for depressive disorders [34-36] and increase medication response in depressed patients [52-57].

## Abbreviations

AOR: Adjusted odds ratio; BMI: Body mass index; BRFSS: Behavioral Risk Factor Surveillance System; CI: Confidence interval; EDS: Elevated depressive symptoms; NHANES: National Health and Nutrition Examination Survey; PHQ: Patient Health Questionnaire; SE: Standard error; VITAL: Vitamins and Lifestyle.

## Sources of financial support

None.

## Competing interests

The authors declare that they have no competing interests.

## Authors' contributions

GZ conducted the data analyses, interpreted the data, and prepared the manuscript. ESF supervised the data analyses and contributed to the manuscript writing. ESF, CL, KJG, JBC, and LSB made critical revisions of the manuscript for important intellectual content. All authors have read and approved the final version of the manuscript.
